# Immunometabolism changes in fibrosis: from mechanisms to therapeutic strategies

**DOI:** 10.3389/fphar.2023.1243675

**Published:** 2023-07-27

**Authors:** Lixiang Feng, Xingyu Chen, Yujing Huang, Xiaodian Zhang, Shaojiang Zheng, Na Xie

**Affiliations:** ^1^ West China School of Basic Medical Sciences and Forensic Medicine, Sichuan University, and State Key Laboratory of Biotherapy and Cancer Center, West China Hospital, and Collaborative Innovation Center for Biotherapy, Chengdu, China; ^2^ Hainan Cancer Clinical Medical Center of the First Affiliated Hospital, Key Laboratory of Tropical Cardiovascular Diseases Research of Hainan Province and Key Laboratory of Emergency and Trauma of Ministry of Education, Hainan Medical University, Haikou, China; ^3^ Department of Pathology, Hainan Women and Children Medical Center, Hainan Medical University, Haikou, China

**Keywords:** immunometabolism, fibrosis, inflammation, therapies, fibroblast

## Abstract

Immune cells are essential for initiating and developing the fibrotic process by releasing cytokines and growth factors that activate fibroblasts and promote extracellular matrix deposition. Immunometabolism describes how metabolic alterations affect the function of immune cells and how inflammation and immune responses regulate systemic metabolism. The disturbed immune cell function and their interactions with other cells in the tissue microenvironment lead to the origin and advancement of fibrosis. Understanding the dysregulated metabolic alterations and interactions between fibroblasts and the immune cells is critical for providing new therapeutic targets for fibrosis. This review provides an overview of recent advances in the pathophysiology of fibrosis from the immunometabolism aspect, highlighting the altered metabolic pathways in critical immune cell populations and the impact of inflammation on fibroblast metabolism during the development of fibrosis. We also discuss how this knowledge could be leveraged to develop novel therapeutic strategies for treating fibrotic diseases.

## 1 Introduction

Fibrosis in various organisms, such as idiopathic pulmonary fibrosis (IPF), hepatic fibrosis, nephrogenic fibrosis, cardiac fibrosis and systemic sclerosis (SSc), might result in organ failure and death if left unchecked. In response to harmful irritants, including pathogens, toxins, oxidative stress, and autoimmune reactions, fibroblasts of multiple origins, such as mesenchymal cells, resident fibroblasts, and trans-differentiation of other cell types, launch the injury healing reactions via remodeling the extracellular environment to repair tissue integrity (Wynn and Ramalingam, 2012). Normally, the fibrotic program is turned off when tissue healing occurs. However, a chronic inflammatory milieu and dysregulated tissue healing caused by persistent tissue injury result in fibrosis by inducing fibroblast activation and excessive extracellular matrix (ECM) protein deposition (Meng et al., 2016). Specifically, sustained TGF-β and pro-inflammatory cytokines promote the differentiation of fibroblasts into myofibroblasts, which are responsible of secreting collagen and other pro-fibrotic factors (Darby and Hewitson, 2007; Travers et al., 2016). In fibrosis, excessively enhanced ECM production causes an increase in organ stiffness and tissue thickening, ultimately leading to organ dysfunction (Herrera et al., 2018).

Activation of immune cells, including macrophages, neutrophils, B cells and T cells, is crucial for tissue damage repair (Arora et al., 2018; Franco et al., 2020). Recent studies indicate that immune cells undergo cellular metabolic reprogramming during the development of various profibrotic diseases, suggesting a crucial role of immunometabolism in fibrosis (Srivastava et al., 2018; Cho et al., 2020). Compared to the homeostatic immune cells that rely on the tricarboxylic acid (TCA) cycle and oxidative phosphorylation (OXPHOS) for efficient energy production, immune cell activation requires increased glycolysis to supply energy and metabolic intermediates (Lam et al., 2016). Additionally, glutamine metabolism and fatty acid oxidation (FAO) can fuel the TCA cycle to support the energy generation in mitochondria. These metabolic alterations play a vital role in regulating immune cell differentiation and the production of inflammatory factors, promoting inflammation (Xu et al., 2021; Pérez and Rius-Pérez, 2022). From the immunometabolism aspect, the aberrant metabolic reprogramming alters the immune cell function, leading to a chronic inflammatory environment and contributing to fibrosis development (Xu et al., 2021; Pérez and Rius-Pérez, 2022). Additionally, the inflammation and immune responses regulate fibroblast metabolism to increase ECM deposition, further aggravating fibrosis (Zhu et al., 2019). Therefore, targeting the abnormal metabolic signaling molecules may provide a practical pathway to slow fibrosis progression.

This review discusses the crucial role of immunometabolism in fibrotic processes and the clinical applications of intervening with metabolic pathways for treating fibrotic diseases.

## 2 Inflammation-driven metabolic reprogramming in fibrotic fibroblasts

**FIGURE 1:**
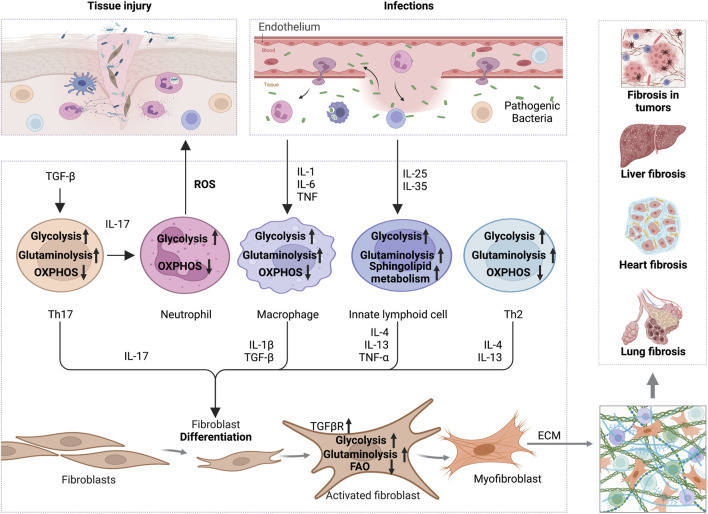
The interplay of inflammation with metabolic reprogramming in the profibrotic environment. TGF-β boosts glycolysis and glutaminolysis in Th17 to promote IL-17 release, which increases ROS secretion by neutrophils and thus promotes tissue injury. On the other hand, increased TGF-βR expression on the surface of fibroblasts promotes glycolysis and glutaminolysis to support ECM production. In addition, pro-inflammatory factors, like IL-1, IL-6, and TNF-α secreted by infected or damaged tissues, enhance the glycolysis and glutaminolysis in macrophages, thus increasing the expression of IL-1β and TGF-β to activate the fibrotic response. Cytokines, including IL-25 and IL-35, increase the glycolysis, sphingolipid metabolism and glutaminolysis in innate lymphocytes, which promote the secretion of IL-4, IL-13 and TNF-α, and activation of myofibroblasts. Th2 cells also secrete IL-4 and IL-13 in a glycolysis and glutaminolysis dependent manner to exert profibrotic effect. Ultimately, the activated myofibroblasts contribute to ECM formation and promote fibrosis in various tissues, including liver, heart, lung and even tumors. Abbreviations: ECM, extracellular matrix; FAO, fatty acid oxidation; IL-1, interleukin-1; IL-4, interleukin-4; IL-6, interleukin-6; IL-10, interleukin-10; IL-13, interleukin-17; IL-17, interleukin-17; IL-25, interleukin-25; IL-35, interleukin-35; ROS, reactive oxygen species; TNF-α, tumor necrosis factor alpha; TGF-β, transforming growth factor-beta; TGFβR,TGFβ receptor.

**FIGURE 2:**
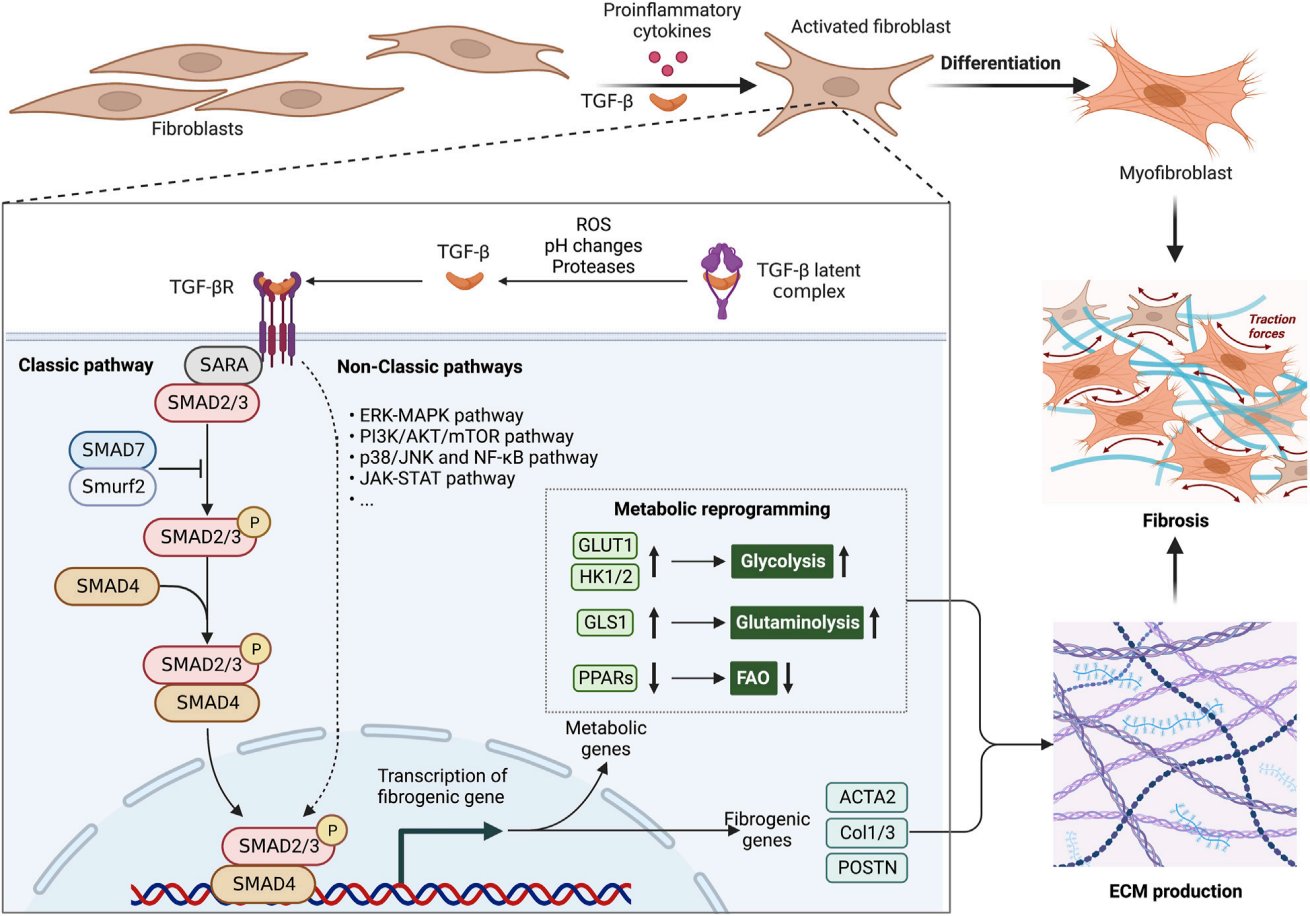
Metabolic alterations are involved in the profibrotic role of TGF-β1 signaling pathway. The aberrant TGF-β1 signaling pathway is an essential pathogenic mechanism in fibrosis. The TGF-β latent complex is activated by proteases, ROS and pH changes in the microenvironment. Activated TGF-β induces the differentiation of fibroblasts into myofibroblasts, by inducing metabolic enzymes in glycolysis and glutaminolysis, as well as inhibiting PPAR signaling-mediated fatty acid oxidation (FAO). These metabolic alterations offer ATP and biosynthetic intermediates for more ECM production. In fibrosis, abnormally ECM production causes an increase in organ stiffness, tissue thickening and lack of mobility, ultimately leading to organ dysfunction. Abbreviations: ERK, extracellular signal-regulated kinase; FAO, fatty acid oxidation; JNK, Jun N-terminal kinase; JAK, Janus kinase; MAPK, mitogen-activated protein kinase; mTOR, mammalian target of rapamycin; NF-κB, nuclear factor kappa-light-chain-enhancer of activated B cells; PI3K, phosphoinositide 3-kinase; ROS, reactive oxygen species; SMAD, suppressor of mothers against decapentaplegic; Smurf2, SMAD ubiquitination regulatory factor 2; STAT, signal transducer and activator of transcription; TGF-β, transforming growth factor-β; TGFβR,TGFβ receptor.

Increasing evidence showed that metabolic reprogramming in activated fibroblasts is an essential feature of fibrosis and its associated diseases (Wang et al., 2019; Wang Q. et al., 2021; Ning et al., 2023). Chronic inflammation induces the metabolic alterations in fibrotic fibroblasts, including glycolysis, amino acid metabolism, fatty acid oxidation (FAO), and fatty acid synthesis (FAS). These metabolic alterations have been implicated in forming ECM and inflammatory environments (Figure 1). Additionally, TGF-β and its associated signaling pathways have been approved to be a key regulator for metabolic reprogramming-driven fibrosis (Jiang et al., 2015; Xie et al., 2015; Wang F. et al., 2023) (Figure 2).

### 2.1 Increased glycolysis in fibrosis

Microenvironmental factors, such as inflammatory factors, lactate and hypoxia, induce the glycolytic reprogramming of fibroblasts during fibrosis. Pro-inflammatory factors released by immune cells, such as PDGF-BB, IL-12, IL-1β and MIP-1β, promote the glycolytic process in fibroblasts. The metabolic switch from OXPHOS to glycolysis can offer more ATP and biosynthetic intermediates, which act as the building blocks for the excessive production of ECM (Kruse and Bornstein, 1975; Im et al., 1976; Xie et al., 2015).

The accumulating lactate in the microenvironment will also boost the glycolysis in stroma cells, promoting ECM deposition and fibrotic process. Activated immune cells with an increased glycolysis release large amounts of lactate, which induces the HIF-1-mediated expression of glycolytic genes, including GLUT1, LDH, and hexokinase (HK), in fibroblasts (Kozlov et al., 2020). Similarly, the elevated level of lactate in the lung tissue of IFP patients due to hypoxia increased the glycolysis inmesenchymal progenitor cells (MPCs) through the HIF-1α pathway. This metabolic reprogramming promotes the differentiation of MPCs into fibroblasts, which further contributes to the exacerbation of lung fibrosis (Yang et al., 2023). While pharmacological and siRNA-mediated suppression of LDH, an enzyme that catalyzes the conversion of pyruvate to lactate, in lung fibroblasts impeded the transition of fibroblast to myofibroblast (Kottmann et al., 2015). Lactate also promotes the migration and accumulation of T cells at the injury sites to create a chronic inflammation environment, which further activate fibroblast to synthesis ECM (Haas et al., 2015). Consistently, higher levels of lactate in the blister fluid of SSc patients was proven to increase collagen production (Taki et al., 2020).

Additionally, increased glycolysis may also fuel the TCA cycle to produce more mitochondrial metabolic intermediates that drive fibrotic progression. Rats with carbon tetrachloride (CCl4)-induced liver fibrosis had higher levels of the TCA cycle metabolites in their serum (Chang et al., 2017). Higher levels of succinate in TGF-β-stimulated lung fibroblasts stabilize HIF-1α to induce glycolysis and decrease FAO, thereby increasing the production of ECM (Xie et al., 2015; Wang Z. et al., 2021). The early intermediates in the TCA cycle, like α-ketoglutarate (α-KG), are crucial precursors for collagen synthesis. Increased glycolysis might promote fibrosis by increasing the α-KG production from the TCA cycle to support the collagen synthesis (Henderson and O'Reilly, 2021). Additionally, the NADH and FADH2 derived from the increased TCA cycle can then supply electrons to the electron transport chain (ETC) and drive OXPHOS, resulting in an elevated production of reactive oxygen species (ROS). ROS can induce TGF-β gene expression in various cell types, including macrophages and fibroblasts (Pociask et al., 2004; Jobling et al., 2006; Liu and Gaston Pravia, 2010). It has been shown that ROS convert inactive latent TGF-β to active formation by oxidizing latency association protein (LAP) (Pociask et al., 2004). Through activating TGF-β pathway, H_2_O_2_ drives epithelial mesenchymal transition (EMT) in lung epithelial cells to promote lung fibrosis (Kume et al., 2023).

### 2.2 Elevated glutaminolysis

In addition to glycolysis, glutaminolysis can also fuel the TCA cycle by glutamate dehydrogenase (GDH)-catalyzed conversion of glutamate into α-KG. Glutaminolysis plays a crucial role in amino acid synthesis, lipid metabolism, and energy production, providing energy and materials for ECM synthesis in fibroblasts. Glutaminolysis enhances glycolysis via the α-KG/mTOR/HIF-1α pathway, which plays an important role in fibrosis. Therefore, elevated glutaminolysis is also viewed as a crucial factor in the development of fibrosis (Gibb et al., 2022).

Inflammation at the site of injury and infection increases glutaminolysis in fibroblasts, providing energy and resources for ECM synthesis and secretion, which in turn promotes fibrosis (Henderson et al., 2020; Qiu et al., 2021). By activating SMAD3 and p38-MAPK signaling pathways, TGF-β1 increases the glutaminase 1 (GLS1) expression and glutaminolysis in myofibroblasts and cardiac fibroblasts (Bernard et al., 2018; Henderson et al., 2020; Chattopadhyaya et al., 2022). Inhibiting glutaminolysis results in a reduce in α-KG and less ECM production in fibroblasts (Henderson et al., 2020). Fibrosis development is slowed by inhibiting the enzymatic activity of GDH with epigallocatechin-3-gallate (EGCG) both *in vitro* and *in vivo* (Karsdal et al., 2020; Yin et al., 2022). Moreover, the transamination of glutamine to glutamate at the first stage of glutaminolysis provides nitrogens for amino acid synthesis and subsequent collagen production (Karsdal et al., 2020). α-KG also increases collagen translation and stability through activating mTOR signaling pathway and promoting collagen proline hydroxylation (Shi et al., 2022). As a result of mTORC1 activation, there is an increase in nutritional absorption, which in turn promotes glutaminolysis by increasing glutamine availability. Additionally, mTORC1 phosphorylates LARP6 to increase collagen expression in fibrosis (Zhang and Stefanovic, 2017).

Furthermore, the TCA cycle intermediates serve as cofactors for epigenetic regulatory enzymes in fibroblasts, such as histone demethylases or histone acetylases. α-KG is a cofactor for Jumonji C domain-containing demethylase 3 (JMJD3), which specifically regulates gene expression by demethylating the trimethylation on histone H3 lysine 27 (H3K27me3) (Tsukada et al., 2006). Increased α-KG derived from glutaminolysis enhances the JMJD3 activity, promoting fibrosis by inducing histone demethylation in particular regulatory regions (Bai et al., 2019; Jia et al., 2019). For example, JMJD3 decreases the accumulation of H3K27me3 at the FRA2 promoter, leading to fibroblast activation in SSc (Bergmann et al., 2018). In a mouse model of obstructive nephropathy, JMJD3 is markedly increased and activated in the kidney with the development of renal fibrosis. JMJD3 activates myeloid fibroblasts by increasing the H3K27 dimethylation in the obstructed nephropathy (An et al., 2023). However, some studies suggest a protective effect of JMJD3 in fibrosis. JMJD3 protects against renal fibrosis by inhibiting fibrotic signals, including TGF-β and Notch signaling (Yu et al., 2021). Consistently, inhibition of JMJD3 in rat hepatic stellate cells has been found to increase the levels of H3K27me3 and fibrosis markers (Jiang et al., 2021). More detailed studies are needed to elucidate whether α-KG regulate the role of JMJD3 in fibrosis in order to provide more strategies for the treatment of fibrosis. Besides, α-KG also modulate histone methylation dynamics of H3K9 and H3K4 mediated by the lysine specific demethylase 1 (LSD1). LSD1 epigenetically activates TGF-β1/Smad3 signaling, which contributes to pulmonary myofibroblast differentiation and fibrosis (Pan et al., 2020; Dong et al., 2022). Additionally, citrate derived from TCA cycle can be converted to acetyl-CoA, which is the substrate for histone acetylation and plays an important role in fibrosis (Wellen et al., 2009).

### 2.3 Reduced lipid metabolism

Fatty acid (FA) metabolism also provides energy to assist cells in adapting to environmental changes (Bartlett and Eaton, 2004; de Carvalho and Caramujo, 2018). In response to inflammation caused by tissue injury and infection, the aberrant FAO and consequent lipid synthesis impair ECM breakdown, contributing to the pathogenesis of fibrotic diseases (Chu et al., 2019; Zhao et al., 2019). It has been found that long-chain fatty acid transporter cluster of differentiation 36 (CD36) interacts with the elements of ECM, suggesting an interplay of fatty acid metabolism and ECM production (Febbraio et al., 2001; Susztak et al., 2005). In rheumatoid arthritis (RA), CD36 expression in fibroblasts is increased by macrophage-derived inflammatory mediators, including interleukin-1β (IL-1β), IL-6 and interferon gamma (IFN-γ) (Ahmed et al., 2008; Komatsu and Takayanagi, 2012). The binding of CD36 to thrombospondin 1 (TSP-1) inhibits the breakdown of ECM (Brown and Ahmed, 2019). Another crosstalk mechanism of lipid metabolism and ECM homeostasis is the TGF-β1/SMAD3-mediated repression of PGC1α or PPARs. The increased MiR-27a in diabetic nephropathy as a result of thylakoid cell damage activates TGF-β/SMAD3 signaling and inhibits PPARγ. The decreased levels of PPARγ result in FAO inhibition and lipid accumulation by reducing the expression of carnitine palmitoyl transferase 1 (CPT1) and acyl-CoA oxidase (ACOX) in fibroblasts, leading to renal tubular interstitial fibrosis (Wang Q. et al., 2018; Zhao et al., 2019). In patients with renal fibrosis, the absence of FAO raises CD36 expression in renal tubular epithelial cells, which in turn increases lipid uptake and intracellular lipid storage, promoting the synthesis and secretion of ECM. The lipotoxicity of excessive lipid accumulation also promotes the development of fibrosis (Herman-Edelstein et al., 2014; Yang et al., 2017). Additionally, fatty acid uptake is also regulated by caveolin-1, a principal component of caveolae, which modulate the surface availability of CD36. In response to bleomycin (BLM), CdCl2, TGF-β and irradiation, the caveolin-1 in fibroblasts is reduced, which concomitantly decreases the surface localization of CD36. Then, the CD36-mediated uptake of fatty acids is diminished, driving a metabolic switch from OXPHOS to aerobic glycolysis in fibroblasts (Ring et al., 2006; Wang et al., 2006; Del Galdo et al., 2008; Castello-Cros et al., 2011).

The disturbed FA metabolism also contributes to malfunction of epithelial cells, which is crucial for the development of pulmonary fibrosis. Alveolar type 2 cells have the most active lipid metabolism among lung epithelial cells, rendering them frequently prone to injury (Barkauskas et al., 2013). Disturbed FA biosynthesis and composition will lead to endoplasmic reticulum (ER) stress by regulating the transportation of folded proteins in lipid droplets. The persistent ER stress due to reduced lipid synthesis can cause damage to lung epithelial cells (Velázquez et al., 2016). These damaged lung epithelial cells create an inflammatory environment and the metabolic reprogramming of fibroblasts, leading to lung fibrosis (Katzen and Beers, 2020). According to the aforementioned evidence, a promising approach for treatment of fibrosis involves targeting lipid metabolic activities, such as FAO and lipid synthesis.

### 2.4 TGF-β mediates the crosstalk between inflammation and metabolic alteration

The profibrotic cytokine TGF-β is a significant part of complicated molecular mechanisms behind fibrosis (Hu et al., 2018). TGF-β mediates the crosstalk between inflammation and metabolic alteration in fibroblasts (Figure 2), which contributes to enhanced ECM synthesis and subsequent fibrosis (Yin et al., 2019; Henderson et al., 2020; Gao and Chen, 2022). During injury tissue repair and infections, activated macrophages and T cells release large amounts of TGF-β (Nakazaki et al., 2021; Zhao et al., 2023). Th17 cells increase the production of TGF-β in response to pro-inflammatory cytokine IL-17A (Lei et al., 2016). Moreover, TGF-β is a key component of senescence-associated secretory phenotype (SASP) factors derived from senescent cells. Cellular senescence is involved in lung fibrosis by releasing SASP factors and recruiting a large number of inflammatory cells into tissue and organs (Goliwas and Deshane, 2020). In addition, senescent cells secrete numerous pro-inflammatory cytokines which may drive the metabolic changes of fibroblasts and promote the synthesis of ECM proteins, contributing to the development of fibrosis (Jun and Lau, 2017; Wiley et al., 2019; Zhang et al., 2019; Blokland et al., 2020). Metabolic reprogramming like increased glycolytic pathway and mitochondrial dysfunction increase reactive oxygen species (ROS) production in senescent fibroblasts, which trigger the secretion of TGF-β (Hassona et al., 2013; Correia-Melo et al., 2016; Wiley et al., 2016). TGF-β/SMAD signaling pathway induces the senescence of adjacent cells in a paracrine manner by upregulating the cell cycle inhibitors p21, p27 and p15 (Muñoz-Espín and Serrano, 2014). In response to TGF-β, TGF-βRI induces the formation and nucleus translocation of SMAD 2/3/4 complex, which activates the transcription of profibrotic and metabolic genes while suppresses antifibrotic targets (Budi et al., 2017). Particularly, TGF-β signaling promotes cell proliferation, fibroblast-to-myofibroblast differentiation, and ECM synthesis, as well as reduces matrix protease secretion in the context of fibroblasts (Chen et al., 2018; Eser and Jänne, 2018). Interestingly, in mouse and human fibrotic lung tissue, cadherin-11 ensures continuous activation of myofibroblasts by linking TGF-β-producing macrophages and TGF-β-activated myofibroblasts (Lodyga et al., 2019).

Besides regulating inflammation, TGF-β induces the metabolic reprogramming of fibroblasts, resulting in an increased glycolysis and glutamine catabolism, as well as enhanced ECM synthesis (Yin et al., 2019; Henderson et al., 2020; Gao and Chen, 2022). TGF-β induces glycolysis and glutaminolysis to boost collagen production in fibroblasts. The expression of glycolytic transporter GLUT1 and metabolic enzyme HK1/2 is increased by TGF-β signaling to promote glycolysis in fibroblasts (Masumi et al., 1993; Yin et al., 2019; Chen et al., 2022). Fibroblasts in IPF have increased HK2, an enzyme that produces glucose 6-phosphate during the initial phase of glycolysis, which increased collagen expression through YAP (Yin et al., 2019). It has also been shown that GLUT1 and HK1/2 are upregulated in keloid fibroblasts, promoting the collagen synthesis and fibrosis development (Vinaik et al., 2020; Wang Q. et al., 2021). TGF-β also induces the glycolytic reprogramming by HIF-1 in hepatic stellate cells (HSCs), which plays an important role in increasing collagen production and finally hepatic fibrosis (Hanna et al., 2013; Mohammad Omar et al., 2022). Additionally, TGF-β1 upregulates glutaminase (GLS) expression in myofibroblasts, thereby increasing glutaminolysis to provide energy for the synthesis of ECM protein (Bernard et al., 2018).

## 3 Immunometabolism controls immune cells in fibrosis

**FIGURE 3:**
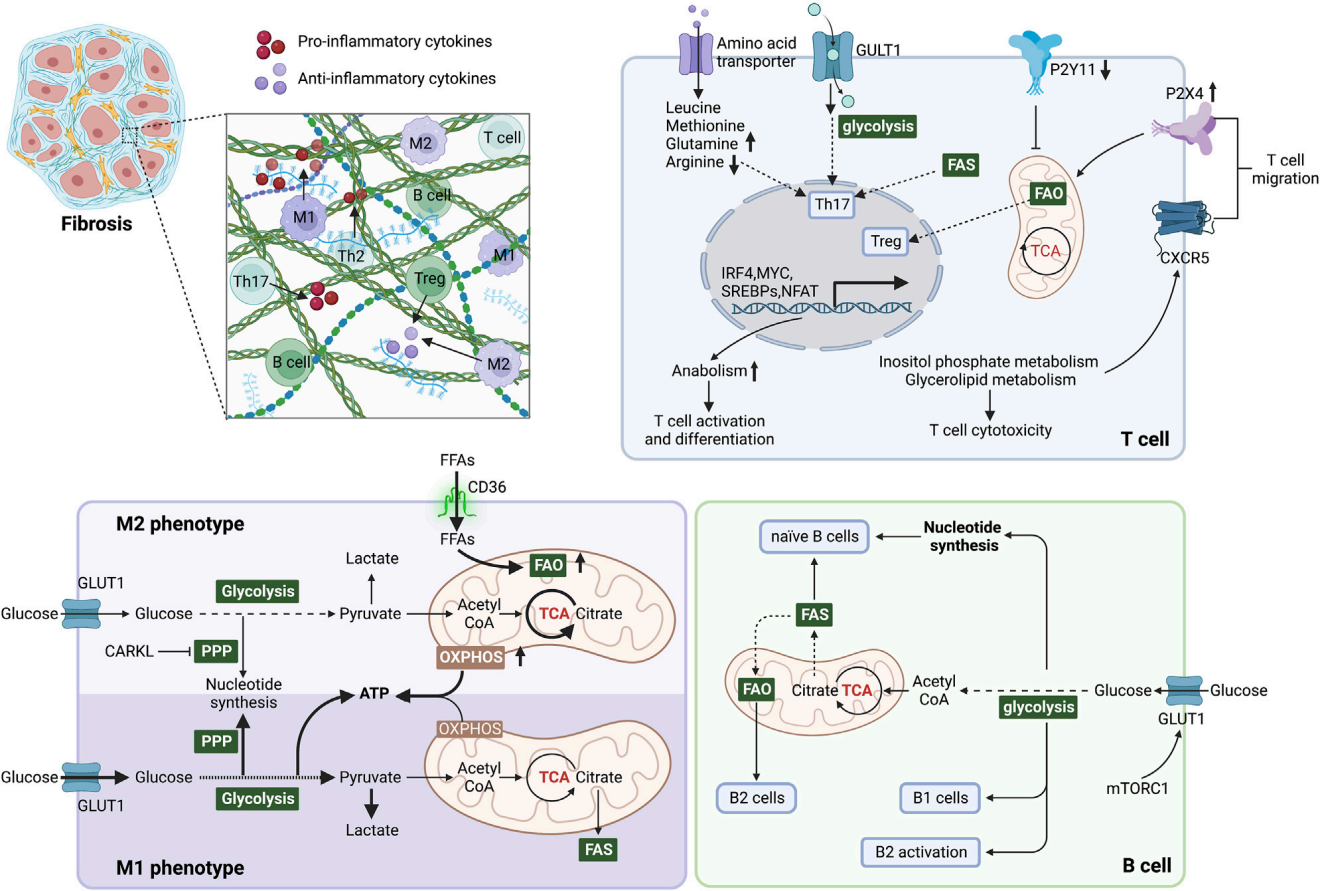
Immunometabolism determines immune cell function and inflammatory microenvironment during fibrosis. In M1 phenotype macrophages, glycolysis is the primary energy supply mode, elevating lactate production and inhibiting OXPHOS in mitochondria. In addition, the pentose phosphate pathway (PPP) is enhanced to generate NADPH, promoting the synthesis and release of inflammatory factors. Elevated FAO and OXPHOS promote macrophage polarization to the M2 phenotype. CARKL inhibits PPP and converts macrophages to the M2 phenotype. Glycolysis, glutaminolysis and lipid synthesis are required for Th17 cell differentiation and function, but in contrast, Treg cells perform catabolism and enhanced FAO to induce mitochondrial oxidative metabolism. Purinergic receptors influence mitochondrial metabolism by sensing local ATP content, which affects T cell migration. Mitochondrial metabolism is blocked by P2Y11 and activated by P2X4. Inositol phosphate metabolism and glycerolipid metabolism promote the migration of T cells by stabilizing the follicle homing receptor CXCR5 on their surface. Naive B cells mainly obtain energy and nucleotide biosynthesis through the TCA cycle and OXPHOS. The survival and function of B1 cells are dependent on glycolysis, whereas splenic B2 cells rely on FAO. During B2 cell activation, elevated GLUT1 expression induced by c-Myc and mTORC1 increases glucose uptake and shifts FAO to glycolysis. After metabolic reprogramming, immune cells regulate the microenvironment by secreting pro/anti-inflammatory factors, thereby affecting fibrosis. Abbreviations: ATP, adenosine triphosphate; CARKL, carbohydrate kinase-like protein; c-Myc, proto-oncogene Myc; FAS, fatty acid synthesis; FFAs, free fatty acids; FAO, fatty acid oxidation; GLUT1, glucose transporter 1; mTORC1, mTOR complex 1; mTOR: mammalian target of rapamycin; PPP, pentose phosphate pathway; TCA, tricarboxylic acid.

Immunometabolism is essential in controlling the immune cell function and activity during fibrotic process (Figure 3). In this section, we review recent advances in the mechanisms underlying metabolic regulation of immune cells, including macrophages, T cells, B cells and neutrophils, as well their role in fibrotic diseases.

### 3.1 Macrophages

Under normal conditions, the inactive macrophages in a quiescent state, called naive macrophages or M0 macrophages, are supplied with ATP mainly by aerobic oxidation of glucose and β-oxidation of fatty acids (Rőszer, 2015; Ramond et al., 2019). In respond to stimulation, metabolic reprogramming drives polarization of macrophages into either pro-inflammatory M1 or anti-inflammatory M2 phenotype, thereby performing a distinct role in fibrosis (Wynn and Barron, 2010; Malyshev and Malyshev, 2015).

**FIGURE 4:**
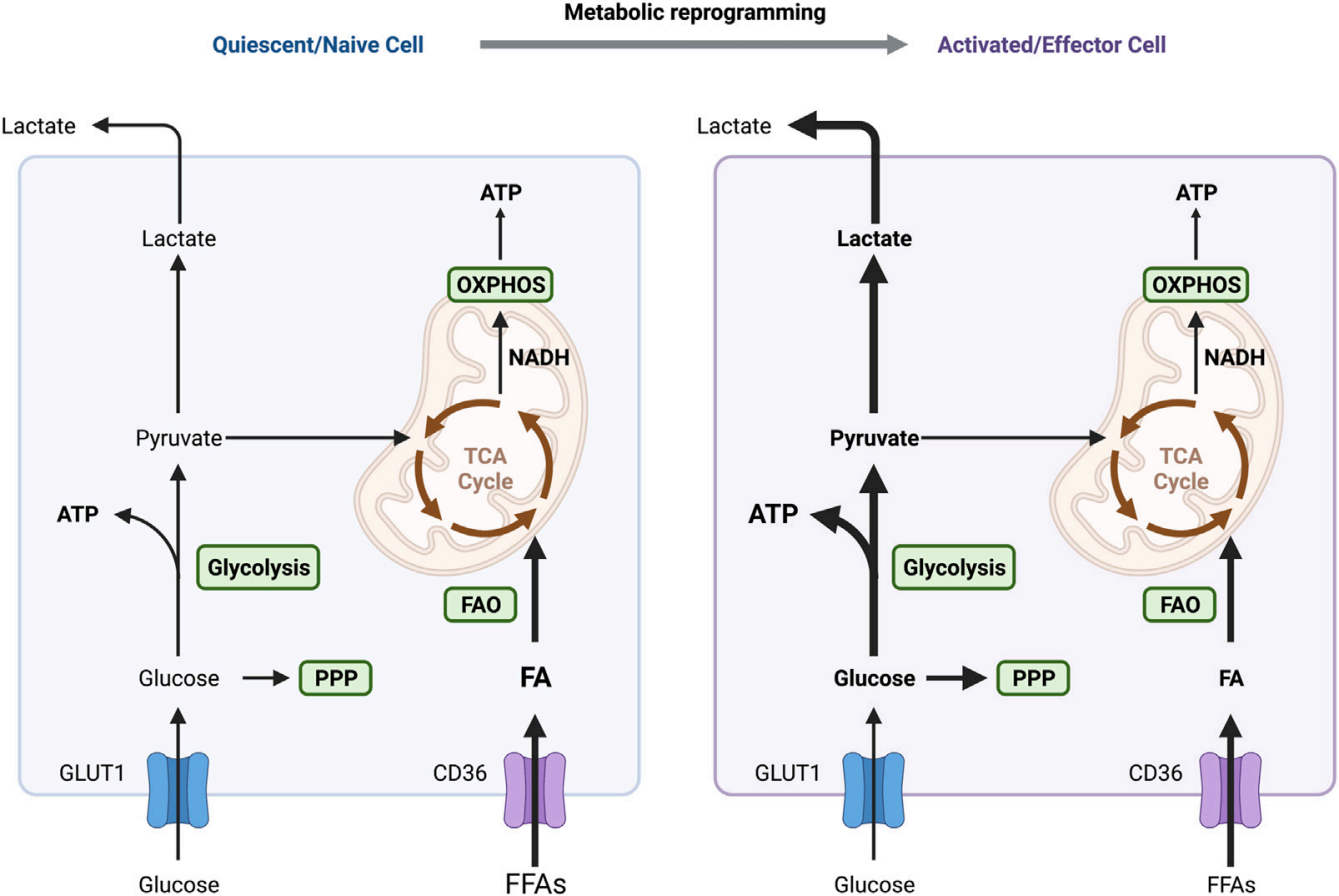
Different metabolic patterns in quiescent and activated immune cells. OXPHOS and FAO are the primary sources of energy in naive and quiescent macrophages and T cells to maintain regular cellular life activities. One of the primary variables in the activation of these immune cells is the metabolic switch between glycolysis and OXPHOS. IFN-γ and LPS-activated macrophages exhibit an increase in aerobic glycolysis, which supplies the energy needed for M1 macrophages to polarize and exhibit pro-inflammatory actions quickly. Similarly, energy supply mode in T cells switches to aerobic glycolysis in response to antigenic stimulation, activating and transforming them into effector cells. Abbreviations: ATP, adenosine triphosphate; FAO, fatty acid oxidation; OXPHOS, oxidative phosphorylation; PPP, pentose phosphate pathway; TCA, tricarboxylic acid.

M1 macrophages undergo a shift from OXPHOS to glycolysis in response to stimulus (Figure 4), like interferon-γ (IFN-γ) secreted by Th1 lymphocytes, exogenous lipopolysaccharide (LPS), exogenous DNA and viral infection. This metabolic reprogramming induces secretion of various cytokines to promote inflammation (Orecchioni et al., 2019; Kang and Kumanogoh, 2020; Lin et al., 2021). M1 macrophages increase the GLUT1 expression for glucose uptake, which supports the enhanced aerobic glycolysis for rapid ATP production (Freemerman et al., 2014). However, the TCA cycle is blocked at the citrate and succinate production steps due to the reduced levels of isocitrate dehydrogenase (IDH) and succinate dehydrogenase (SDH) during M1 polarization (Jha et al., 2015; Corcoran and O'Neill, 2016). The accumulation of cellular lactate, citrate and succinate then regulate their energy metabolism by increasing the levels of intracellular PGE2, ROS, and NO production (El Kasmi and Stenmark, 2015; Corcoran and O'Neill, 2016). Succinate supports the, ETC and ROS generation (El Kasmi and Stenmark, 2015). Succinate or ROS can maintain the stability of HIF-1α, which enhances glycolysis by increasing GLUT1-mediated glucose uptake and LDH-dependent lactate production, driving M1 macrophage polarization (Wang T. et al., 2017; Liu et al., 2017). Iron metabolism also regulates the polarization of M1 macrophages. Excess iron frequently activates M1 polarization and induces the expression of inflammatory factors, such as TNF-α and IL-12 (Sindrilaru et al., 2011). Notably, M1 instead of M2 polarization occurs almost immediately after upregulation of transferrin receptor 1 (TfR1), a membrane receptor that uptake of circulating transferrin-bound iron, and downregulation of ferroportin, the only known iron exporter (DeRosa and Leftin, 2021; Ho et al., 2022).

The metabolic reprogramming is crucial for the production of inflammatory factors. ROS can enhance the DNA binding ability of p65 and increase the NF-κB-mediated expression of inflammatory factors, such as IL-1β and IL-6, which contribute to the polarization of macrophages toward the M1 phenotype (Genard et al., 2017). HIF-1α also promotes the metabolic reprogramming of M1 macrophage polarization and subsequent inflammatory reactions (Corcoran and O'Neill, 2016). In the process of M1 macrophage polarization, HIF-1α upregulate the expression of IL-1β (Tannahill et al., 2013). Additionally, pentose phosphate pathway (PPP) is a critical pathway of high flux glycolysis supporting inflammatory macrophage biosynthesis. The elevated NADPH generation from PPP supports fatty acid synthesis for the production of inflammatory mediators, such as leukotrienes and prostaglandins (Forrester et al., 2020). The main function of classically activated M1 macrophages is to combat acutely harmful infections by producing pro-inflammatory cytokines, including IL-1β, IL-2, IL-6, IL-12, IL-23, TNF-α, and chemokines like nitric oxide (NO) and ROS. However, the excessive amount of pro-inflammatory cytokines secreted by M1 macrophage promotes fibrotic disease during chronical tissue repair (Wick et al., 2010; Xu et al., 2012). For example, TGF-β and pro-inflammatory cytokines like IL-1β can induce fibroblast activation and differentiation into myofibroblasts, leading to overproduction of fibrogenic genes and ECM (Gieling et al., 2009; Gasse et al., 2011; Upagupta et al., 2018; Lv S. L. et al., 2021).

M0 macrophages polarize into the M2 phenotype, which facilitate the healing process after tissue damage in exposure to IL-4, IL-10, IL-13, TGF-β, and glucocorticoids (Stein et al., 1992; Rőszer, 2015; Ishida et al., 2023). The energy of anti-inflammatory M2 macrophages is mainly provided by OXPHOS and FAO (Liu et al., 2017; Van den Bossche et al., 2017). In response to IL-4 and IL-13, STAT6 together with peroxisome proliferator activated receptor-γ coactivator-1β (PGC-1β) stimulates PPARγ signaling pathway and FAO, which polarizes the macrophages toward the M2 phenotype (Olefsky and Glass, 2010). Increased CD36 expression and enhanced FAO in M2 macrophages produce more acetyl CoA, which can enter the TCA cycle to ensure the production of ATP from OXPHOS (Chowdhury et al., 2019; Wilson et al., 2019). In addition, IL-6, IL-13 and IL-14 stimulate HIF-2α to drive metabolic reprogramming and polarization of macrophages to M2 phenotype (Schultze et al., 2015). HIF-2α can subsequently activate PPAR-γ to induce the expression of arginase-1 (Arg-1), which catalyzes the urea cycle and increases NO production (Wang Q. et al., 2018). In terms of iron metabolism, in contrast to M1 macrophages, the expression of iron metabolism-related proteins, such as ferritin and TfR1, is decreased during the polarization of M2 macrophages (DeRosa and Leftin, 2021). The uptake of ω-6 and ω-3 unsaturated fatty acids facilities the synthesis of pro-resolving mediators, such as maresins, protectins, lipoxins, and resolvins (Serhan and Levy, 2018). They bind to ALX/FPR2, GPR18, and chemerine of macrophages, promoting the transition of macrophages into M2 phenotypes while inhibiting the production of pro-inflammatory molecules (Serhan, 2014; Chiang and Serhan, 2017). Pro-resolving chemicals produced by M2 macrophages, including TGF-β and IL-10, inhibit inflammation by enhancing tissue repair and remodeling (Cui et al., 2023).

Metabolic reprogramming-mediated macrophage polarization promotes fibrotic diseases. It has been shown that M2 macrophages ensure profibrotic function in multiple ways. Anti-inflammatory M2 macrophages promote the progression of pulmonary fibrosis by secreting platelet-derived growth factor (PDGF), IL-1β, and TGF-β (Goda et al., 2020). M2 macrophages promote ECM production by recruiting fibroblasts, inducing EMT and increasing L-arginine metabolism for collagen synthesis (He et al., 2019; Wang et al., 2020). Notably, macrophages play a dual role in liver fibrosis, i.e., M1 macrophages promote liver fibrosis while M2 macrophages attenuate liver fibrosis (Kisseleva and Brenner, 2021; Wang Z. et al., 2023). The metabolic reprogramming is required for M1 macrophages to produce inflammatory mediators, which induce the differentiation of HSCs into myofibroblasts for excessive ECM generation (Bility et al., 2014; Xie et al., 2021). In addition, during the inflammatory phase, M1 microphages increase the production of tissue inhibitor of matrix metalloproteinase (TIMP) to inhibit ECM degradation (Mahalanobish et al., 2020). However, M2 macrophages degrade ECM during the anti-inflammatory phase by secreting MMPs (i.e., MMP1, MMP12, MMP9), thus promoting fibrosis resolution (Ramachandran et al., 2012; Zhao et al., 2022). Oxidative stress is a key event in pulmonary fibrosis due to inflammation. ROS promote the conversion of macrophages from the M1 phenotype to the M2 phenotype, thereby contributing to the development of pulmonary fibrosis (Kurundkar and Thannickal, 2016). NADPH oxidases (NOXs)-derived ROS are responsible for monocyte-to-macrophage differentiation by activating MAPK/JNK and ERK signaling, which affecting M2 macrophage polarization (Xu et al., 2016). Mitochondrial ROS in M1 macrophages induce STAT6-dependent JMJD3 expression, which increases the transcriptional activation of M2 genes (He et al., 2016).

### 3.2 T cell

Infiltrated T cells participate in wound healing and tissue repair by promoting the activation of HCSs, formation of myofibroblasts and inflammation (Pellicoro et al., 2014; Koyama and Brenner, 2017; Rao et al., 2021). Metabolic reprogramming is required to meet the growth, proliferation and effector functions of T cells during fibrotic responses (Figure 4).

To maintain aerobic glycolysis, T cells increase GLUT1 expression and the glucose uptake through the CD28-dependent PI3K/Akt signaling pathway (Frauwirth et al., 2002). It has been found that mTOR-activated HIF-1α increased the expression of glycolytic enzymes, including HK and phosphofructokinase and pyruvate dehydrogenase kinase (PDK) in T cells (Düvel et al., 2010; Waickman and Powell, 2012). The mTORC1 signaling pathway in T cells also promotes fibroblast activation, contributing to the development of renal interstitial fibrosis (Chen et al., 2016). Naive T cells from RA patients have impaired glycolytic flux as a result of elevated glucose-6-phosphate dehydrogenase (G6PD) expression. Overexpression of G6PD shunts glucose into PPP for NADPH production, which eliminates the ROS and promotes the differentiation of T cells to pro-inflammatory Th1 and Th17 cells in RA (Yang et al., 2016). Alterations in the PPP pathway also render T cells sensitive to necrosis and significantly promote inflammatory responses in patients with systemic lupus erythematosus (Gergely et al., 2002). Upon T cell activation, enhanced aerobic glycolysis leads to activation of JAK-STAT1 pathway and secretion of IFN-γ, which further increases the classical IFN-stimulated gene expression and functions as a crude fibrogenic agent in fibrosis development (Villarino et al., 2017).

Amino acid metabolism, including glutamine, arginine, serine and glycine, is critical in T cell proliferation and differentiation (Wang et al., 2011; Kishton et al., 2016; Ma et al., 2017). Amino acids serve as the basic structure for protein synthesis, one source for providing energy and upstream regulators of mTORC1 (Rebsamen et al., 2015; Huang et al., 2021). It has recently been found that amino acids are converted into α-ketoglutarate, which can fuel the TCA cycle to produce energy and metabolites required for T cell activation (Newsholme et al., 1985; Wang and Green, 2012). Amino acids serve as upstream regulators of mTORC1 to modify intracellular ATP levels and energy metabolism (Shi et al., 2019; Huang et al., 2021). The disrupted amino acid metabolism exacerbates CCl4-induced liver fibrosis by promoting the release of fibrotic mediators from T cells, including TGF-β, IL-4, IL-6 and IL-17 (Yoshizaki et al., 2010; Shan et al., 2016). T cell receptor signaling can increase the expression and cell membrane localization of amino acid transporter (Nakaya et al., 2014). SLC7A5, a transporter for branched neutral amino acids such as leucine and phenylalanine, is highly increased in activated CD8^+^ T cells in response to antigen stimulus (Sinclair et al., 2013). CD4^+^ T cells are unable to proliferate and differentiate into Th1 and Th 17 cells in SLC7A5-deficient mice (Nakaya et al., 2014).

Iron homeostasis is also critical in the activation of T cells and in the regulation of their functions (Bonaccorsi-Riani et al., 2015). Reduced numbers of mature T cells were found in H-ferritin knockout mice, indicating that ferritin-stored iron is important for the survival of T lymphocytes (Vanoaica et al., 2014). During early T cell activation, mediated by IL-2, T cells take up iron via TfR1 to assist T cell activation (Macedo et al., 2004). Notably, iron inhibits Th1 cell differentiation, IFN-γ expression via upregulating of T cell immunoglobulin and mucin containing protein-3 (TIM-3) (Pfeifhofer-Obermair et al., 2021). Interestingly, some iron oxide nanoparticle-based adjuvants can instead promote the induction of Th1 and Th17 activation and immune responses (Neto et al., 2018)]. Similarly, iron has been shown to inhibit Th2 cell differentiation and immune function (Wang S. et al., 2021). TfR1 expression is higher in Treg than in CD4^+^ T cells, which leads to more iron translocation into Treg cells, and this in turn mediates Treg death (Feng et al., 2021).

Compared to Th1 and Th17 cells with high levels of glycolysis and low levels of FAO, Tregs with a low level of GLUT1 have a higher rate of lipid oxidation to fuel the mitochondrial, ETC for proliferation and differentiation (Michalek et al., 2011). Of note, FAO-driven Tregs are essential in regulating the development of fibrosis. In a diabetic mouse model, the ketogenic diet (KD), a very low-carbohydrate, high-fat diet, inhibits the mitochondrial respiration, FA synthesis and FAO in Tregs (Tao et al., 2021). This metabolic alteration impairs the Treg expansion and function, promoting cardiac fibroblast activation and interstitial fibrosis (Tao et al., 2021). Similarly, depletion of Tregs cells increases Th1/Th2 cytokine and collagen deposition, contributing to the diphtheria toxin-induced lung fibrosis (Moyé et al., 2020).

### 3.3 B cell

**FIGURE 5:**
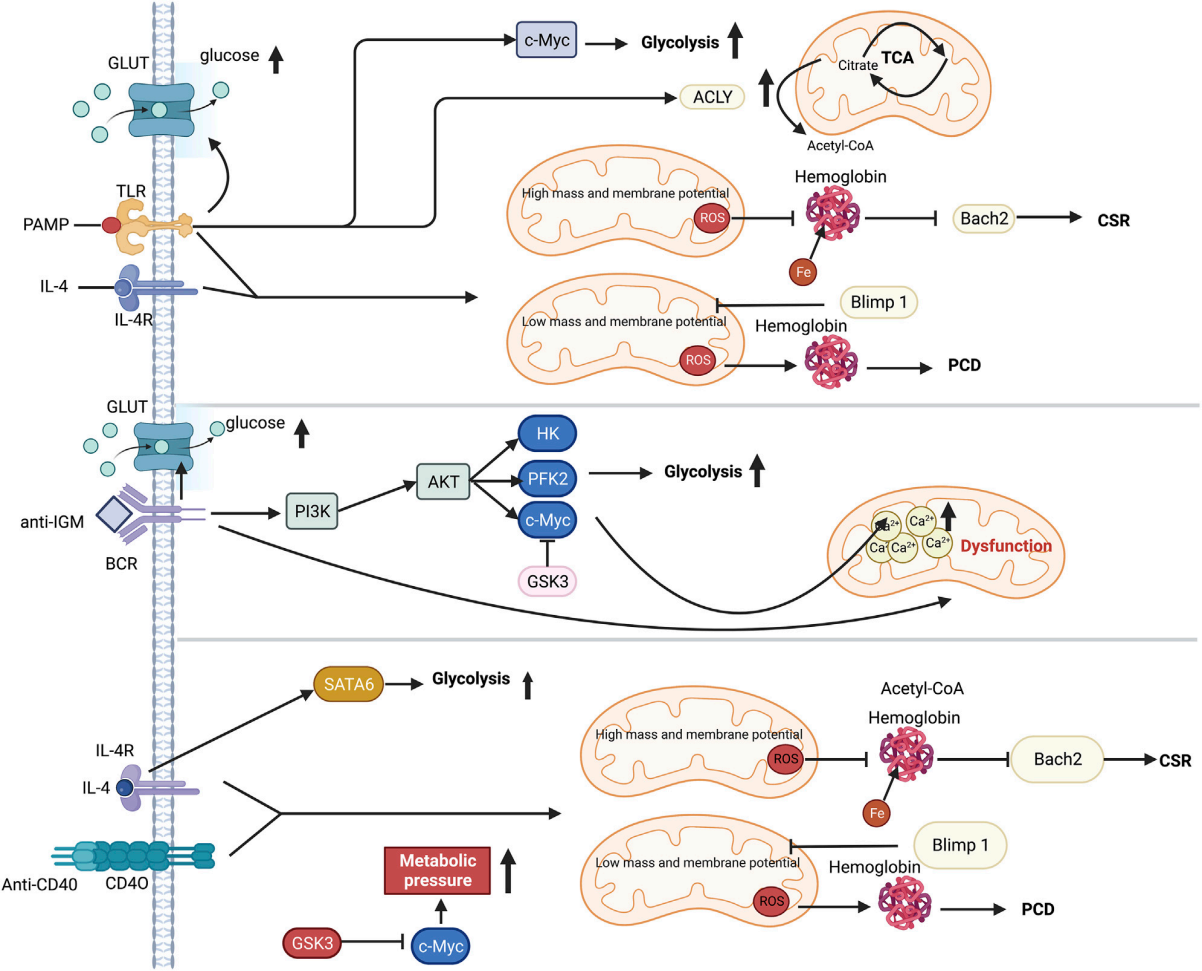
Metabolic reprogramming in B cell following TI or TD stimulation. Selected metabolic process is induced by TI or TD stimulation. While PI3K and GSK3 coordination is necessary for anti-IgM-induced metabolic change, T cell dependent antigen (TD-Ag) results in an increase in c-Myc-dependent glycolysis. The cytokine IL-4 can increase glycolysis by activating STAT6. Mitochondria perform a significant role in distorting the fates of B cells after antigen stimulation. Co-stimulation signal is essential for preserving B cells since excessive mitochondrial respiration during single-BCR stimulation results in Ca2^+^ buildup and ultimately leads to death. The production of mitochondrial ROS, which in turn regulates either Blimp1 or Barch2, two crucial transcriptional regulators of plasma development and differentiation, has an impact on hemoglobin stability. Abbreviations: ACLY, ATP-citrate lyase; ATP, adenosine triphosphate; AKT, protein kinase b; BCR, B cell receptor; CSR, class-switch recombination; c-Myc, proto-oncogene Myc; GLUT, glucose transporter; GSK3, Glycogen synthase kinase-3; HK, hexokinase; IL-4, interleukin-4; IL-4R, interleukin-4 receptor; IGM, immunoglobulin M; PCD, plasma cell differentiation; PAMP, pathogen-associated molecular patterns; PFK2, phosphofructokinase 2; PI3K, phosphatidylinositol 3-kinase; STAT6, signal transducer and activator of transcription 6; TLR, toll like receptor.

The altered immunometabolism in B-cell is essential in fibrotic diseases such as the autoimmune disease systemic lupus erythematosus (SLE) (Oleinika et al., 2019; Lee et al., 2021). FAO is the primary energy supply mode in inactivated germinal center B cells (Weisel et al., 2020). Multiple metabolic pathways are activated to provide materials and energy for the proliferation and differentiation of B cells following antigen recognition (Woodland et al., 2008; Caro-Maldonado et al., 2014; Cyster and Allen, 2019). In T cell-dependent (TD) B cell activation, IL-4/STAT6 axis induces the expression of GLUT1 to increase glycolysis (Dufort et al., 2007; Cyster and Allen, 2019). T cell-independent (TI) antigen stimulates TLR4-PI3K-AKT signaling pathway, which also promotes the GLUT1-mediating glucose uptake and subsequent glycolysis (Caro-Maldonado et al., 2014; Jayachandran et al., 2018). Moreover, HIF-1α is activated by ERK-STAT3 and NF-kB pathways following BCR and TLR stimulation (Meng et al., 2018). HIF-1α together with c-Myc induces the expression of GLUT1 and glutamine transporter, increasing glycolysis and glutaminolysis (Caro-Maldonado et al., 2014). Additionally, ATP-citrate lyase (ACLY) is increased by LPS to promote fatty acid synthesis, including FFAs, neutral and acidic phospholipids and cholesterol, supporting the proliferation and differentiation of B cells (Dufort et al., 2014). Furthermore, it has been recently found that iron is a key element in promoting B cell proliferation and antibody production after TD or TI stimulation (Jiang et al., 2019). Iron promotes B-cell proliferation by inducing histone 3 lysine 9 demethylation at the cyclin E1 promoter, thereby increase cyclin E1 expression (Jiang et al., 2019). Similarly, lactoferrin (LF), a pleiotropic iron-binding glycoprotein, stimulates TGF-β signaling to produce IgA and IgG2b in B cell (Jang Y. S. et al., 2015). B cells quickly die following a mitochondrial reprogramming initiated by BCR stimulation alone. However, mitochondrial dysfunction is prevented by TLRs signaling or CD40 co-stimulation (Akkaya et al., 2018). Upon co-stimulation by IL-4 and CD40L, the metabolic stress due to elevated glycolysis and mitochondrial biosynthesis leads to ROS accumulation, which could be counteracted by GSK3 (Jellusova et al., 2017) (Figure 5). The production of ROS by the mitochondria following metabolic reprogramming is related to mitochondrial mass and membrane potential (Echtay et al., 2002; Jang K. J. et al., 2015). Low mitochondrial mass and membrane potential carry out plasma cell differentiation (PCD) in B cells, and high mitochondrial mass and membrane potential drive class switching recombination (CSR) (Jang K. J. et al., 2015) (Figure 5). CSR is a process of molecular rearrangement in which a group of downstream C_H_ genes exchange genes encoding the heavy chain constant region C_H_ in B cells (Chi et al., 2020). Therefore, alterations of C_H_ exon clusters result in production of IgG, IgA, or IgE, in which the antigen-binding variable region is unaltered (He et al., 2016).

Metabolic switch is essential for plasma cell differentiation and antibody production. Plasmablasts consistently express Blimp1, XBP-1 and IRF, which promote the differentiation of mature B cells into plasma cells and B memory cells, as well as induce antibody production (Aronov and Tirosh, 2016; Tellier et al., 2016; Cyster and Allen, 2019). It has been found that plasma cells in the intestine had increased glycolysis and OXPHOS compared to neoplastic B cells (Kunisawa, 2017). The antibody production in plasma cells requires increased protein synthesis, elevated number of ER and related organelles to provide more anabolic raw materials (Manz and Radbruch, 2002). ACLY serves as a link between glucose metabolism and lipid synthesis, which enhances ER activity depending on lipid synthesis (van Anken et al., 2003). Activated ACLY induces the glucose incorporation into biosynthesis of lipids, such as phosphatidylcholine (PC) and ceramide (CM), which are required for ER expansion in B lymphocytes. Inhibiting ACLY-mediated glucose-dependent *de novo* lipogenesis will impair the plasma cell proliferation and differentiation, as indicated by reduced differentiation markers, CD138 and Blimp-1 in response to LPS stimulation (Dufort et al., 2014). Vitamin B1 is an essential coenzyme for the maintenance of pyruvate dehydrogenase (PDH) activity in the TCA cycle and is crucial in the synthesis and secretion of antibodies by plasma cells (Purroy et al., 2020). Long-lived plasma cells and short-lived plasma cells have different metabolic patterns (Tarlinton et al., 2008). Long-lived plasma cells exhibit higher levels of autophagosomes and surface amino acid transport protein CD98 (Lam and Bhattacharya, 2018). Long-lived plasma cells use glucose for protein glycosylation modification in the absence of long-term antigenic stimulation and metabolic stress. After being re-stimulated with pathogen antigens and autoantigen, long-lived plasma cells take up large amounts of glucose for pyruvate-dependent respiration (McHeyzer-Williams and McHeyzer-Williams, 2005; Jang et al., 2016; Zeng et al., 2020). Mice lacking mitochondrial pyruvate carrier 2 (Mpc2), a protein that transports pyruvate from matrix to mitochondria, have a shorter plasma cell lifetime and release less antigen-specific antibodies (Bertolotti et al., 2010; Lightman et al., 2019).

Metabolic reprogramming of B cells is associated with the development of fibrotic diseases. Disrupted formation of autoantibodies serves as a primary pathogenic factor for SLE, a systemic autoimmune disease (Spada et al., 2015). It has been observed that overactivation of PI3K/AKT/mTOR signaling pathway and metabolic reprogramming occur in B cells of SLE patients (Wu et al., 2007; Jones et al., 2016). Inhibition of mTORC1 dramatically reduces autoantibody production, which improves the SLE in mice (Jones et al., 2016; Geier and Perl, 2021). When mTORC1 is suppressed, the expression of immunoglobulin-binding protein (BiP) and other proteins necessary for protein synthesis is decreased, leading to reduced antibody production (Jones et al., 2016). In addition to harming joints, numerous other organs and tissues are also affected by the chronic inflammatory autoimmune illness known as RA (Yang et al., 2019). Similarly, EZH2-driven plasmablast differentiation and generation of autoantibody in RA patients are caused by overactivation of mTORC1 and methionine-dependent spleen tyrosine kinase (Syk) pathways (Zhang et al., 2020). In addition, disrupted lipid or/and glucose metabolism in B cells is frequently observed in metabolic illnesses, eventually leading to fibrosis (Brittain et al., 2016). For example, B cells in obese individuals produce pro-inflammatory factors, like IL-2, IFN-γ, TNF-α and IL-10, compared to wasting individuals. These cytokines make adipose and other tissues susceptible to chronic tissue inflammation in obesity, which can lead to systemic metabolic disorders linked to fibrosis (DeFuria et al., 2013; McLaughlin et al., 2017; Wijngaarden et al., 2022). On the other hand, B cells polarize into regulatory B cells that constitutively secrete IL-10 to control ongoing chronic inflammation in the adipose tissue milieu, which is mediated by saturated FFAs derived from adipocytes (Eguchi et al., 2012).

## 4 Targeting metabolic dysregulation for fibrosis therapy

The metabolic pathways, including glycolysis, FAS, FAO, are the main targets for fibrosis prevention and treatment. Inhibition of key enzymes and regulatory signaling pathways in glycolytic process has been shown to effectively prevent fibrosis by reducing pro-inflammatory immune cell activation and ECM production from fibroblasts. In addition, numerous clinical or preclinical studies have shown that enhancing FAS and FAO by inducing PPAR activity promotes the anti-inflammatory and anti-fibrotic cell phenotype, providing a viable strategy for antifibrotic therapy. On this occasion, we summarize the strategies for targeting metabolic changes in immune and stroma cells for antifibrotic therapy (Table 1).

**TABLE 1:** Targeting metabolic dysfunction for antifibrosis.

Targets	Drugs	Metabolic alterations	Diseases	Clinical phase	References
AMPK	Metformin	Glycolysis↓	Pulmonary fibrosis, renal fibrosis, liver fibrosis	NCT02234440 FDA-approved for type 2 diabetes	Cheng et al. (2021)
		TGF-β1/Smads↓			
		NF-κB↓			
		STAT3↓			Han et al. (2021)
		PGC1α↓			
		ROS↓			
	PXL-770	FAO↑	NASH	NCT03763877 Phase 2	
		OXPHOS↑			
		Glycolysis↓			
		DNL↓			
		TIMP↑			
		TGF-β1↓			
	O-304	Cardiac glucose intake↑	Cardiac fibrosis	Phase 2	Steneberg et al. (2018)
		Glycolysis↓			
		Cardiac glycogen↓			
	Dapagliflozin	Glycolysis↓	Cardiac fibrosis	Preclinical	Tian et al. (2021)
		TGF-β1/Smads↓			
		EndMT↓			
	Resveratrol	SIRT1↑	Liver fibrosis	NCT02030977 Phase 2/3	
		FAS↓			
		FAO↑			
	Spermidine	FAO↑	Colitis	Preclinical	Liu et al. (2020)
		OXPHOS↑			
CPT1	C75	FAS↓	Renal fibrosis	Preclinical	Kang et al. (2015)
		FAO↑			
		OXPHOS↑			
CPT1β and CD36	MCC950	FAO↑	Cardiac fibrosis	Preclinical	Chen et al. (2019)
		OXPHOS↑			
		Glycolysis↓			
FXR	GW4064	YAP phosphorylation↓	Renal fibrosis	Preclinical	Kim et al. (2019)
		Glycolysis↓			
	EDP-305	OXPHOS↑FAS↓	Renal fibrosis, NASH	Phase 2 NCT03421431	Li et al. (2019)
		FAO↑			
GLP1R	Exenatide	AKT/mTOR↓	Cardiac fibrosis	Phase 3 NCT02251431	
		Glycolysis↓			
GPR84 and GPR40	PBI-4050	FAO↑	Pulmonary, skin fibrosis, renal fibrosis	Phase 2	Gagnon et al. (2018)
		OXPHOS↑			Khalil et al. (2019)
		FAS↓			Thibodeau et al. (2019)
	DS-1558	cAMP/PKA↓	Diabetes mellitus 2	Preclinical	Takano et al. (2015)
		FAO↑			
		OXPHOS↑			
Glucokinase	Hesperidin	Glycolysis↓	Liver fibrosis	NCT03377140	
GSK3	Insulin	OXPHOS↑	Liver fibrosis	Phase 2 NCT03377140	
		FAS↓			
Hexokinase	2-DG	Glycolysis↓	Pulmonary fibrosis, renal fibrosis	Preclinical	Xie et al. (2015)
					Ding et al. (2017)
	3-Bromopyruvate	Glycolysis↓	Pulmonary fibrosis	Preclinical	Xie et al. (2015)
HMG-CoA reductase	Pravastatin	TGF-β1/Smads↓	Skin fibrosis	Phase 2 NCT01268202	
		Glycolysis↓			
		Gluconeogenesis↑			
Leukotriene receptor and 5-lipoxygenase	Tipelukast	Glycolysis↓	Pulmonary fibrosis	Phase 2 NCT02503657	
		ROS↓			
PFKFB3	3PO	Glycolysis↓	Pulmonary fibrosis	Preclinical	Xie et al. (2015)
Phosphodiesterase	Pentoxifylline	TGF-β1/Smads↓	Renal fibrosis	Phase 4 NCT03664414	
		Glycolysis↓			
PDK1	Dichloroacetate	Glycolysis↓ mtROS	Pulmonary fibrosis	Preclinical	Goodwin et al. (2018)
PGK1	NG52	Glycolysis↓ mtROS	Cardiac fibrosis	Preclinical	Lu et al. (2023)
PKM2	Shikonin	Glycolysis↓	Renal fibrosis	Preclinical	Ding et al. (2017)
	Celastrol	Glycolysis↓	Liver fibrosis	Preclinical	Fan et al. (2022)
		FAO↑			
		OXPHOS↑			
	TEPP-46	Glycolysis↓	Renal fibrosis	Preclinical	Liu et al. (2021)
PPARα	Ciprofibrate	FAO↑	Pulmonary fibrosis	Approved in the United Kingdom for lowering lipid levels	Oruqaj et al. (2015)
		OXPHOS↑			
		ROS↓			
	Fenofibrate	FAO↑	Liver fibrosis	Preclinical	Lakhia et al. (2018)
	Fenofibrate	FAO↑	Renal fibrosis	FDA approved for lowering lipid levels	Kang et al. (2015)
	WY-14643	FAO↑	Pulmonary fibrosis	Preclinical	Oruqaj et al. (2015)
		Glycolysis↓			
		ROS↓			
PPARα and PPARγ	Troglitazone	FAO↑	Renal fibrosis Liver fibrosis	FDA approved for type 2 diabetes	Routh et al. (2002)
		FAS↓			Xiang et al. (2021)
PPARα and PPARδ	Elafibranor	FAO↑	Liver fibrosis	Phase 3 NCT02704403	
		FAS↓			
PPARγ	Dioscin	FAO↑	Intestinal fibrosis	Preclinical	Wu et al. (2021)
		Glycolysis↓			
	Caffeine plus chlorogenic acid	FAO↑	Liver fibrosis	Phase 2 NCT02704403	
		AS↓			
	Ciglitazone	TGF-β1/Smads↓	Pulmonary fibrosis	Preclinical	Burgess et al. (2005)
		Glycolysis↓			
		ROS↓			
	15d-PGJ2	FAO↑	Pulmonary fibrosis	Preclinical	Burgess et al. (2005)
		TGF-β1/Smads↓			
		Glycolysis↓			
		ROS↓			
	Liothyronine	Glycolysis↓	Pulmonary fibrosis	Preclinical	(Yu et al., 2018)
		Mitochondrial damage↓ mtROS			
PPARγ, STAT3 and pFOXO1	Pioglitazone	Glycolysis↓	Pulmonary fibrosis	Preclinical	Calvier et al. (2017)
		Mitochondrial damage↓ mtROS			

Abbreviations: AMPK, 5′-AMP-activated protein kinase; CPT1, carnitine O-palmitoyl transferase 1; DNL, *de novo* lipogenesis; EndMT, endothelial-to-mesenchymal transition; FXR, farnesoid X receptor; GLP1R, glucagon-like peptide 1 receptor; GPR40, G-protein-coupled receptor 40; GPR84, G-protein-coupled receptor 84; GSK3, glycogen synthase kinase-3; HMG-CoA, 3-hydroxy-3-methyl-glutaryl-CoA; NASH, non-alcoholic steatohepatitis; PDK1, pyruvate dehydrogenase kinase 1; PFKFB3, 6-phosphofructo-2-kinase/fructose 2,6-bisphosphatase 3; pFOXO1, phosphorylated forkhead box protein O1; PGC1α, peroxisome proliferator-activated receptor-gamma coactivator 1alpha; PKM2, pyruvate kinase muscle isozyme M2; PPAR, peroxisome proliferator-activated receptor; STAT3, signal transducer and activator of transcription 3; 3PO, 3-(3-pyridinyl)−1-(4-pyridinyl)−2-propen-1-one; 2-DG, 2-deoxyglucose.

Given the important role of glucose metabolism in producing ECM, targeting glycolysis for antifibrotic therapy has yielded some positive results. For example, inhibiting glycolysis by 2-DG, an inhibitor of hexokinase2 (HK2), or 3PO, a small-molecule inhibitor of PFKFB3, prevents the conversion of lung fibroblasts to myofibroblasts and reduces collagen synthesis (Xie et al., 2015; Hu et al., 2020). Inhibition of glycolysis by 2-DG and shikonin attenuates the extent of renal fibrosis in mice. Impaired glycolysis in renal fibroblasts results in reduced expression of fibrosis markers, including α-SMA and fibronectin, elevated intracellular pH and decreased lactate accumulation (Ding et al., 2017). In addition, plant-derived triterpene celastrol inhibits glycolysis in macrophages by changing the conformation of pyruvate kinase isozymes M2 (PKM2) and inhibiting PKM1. This metabolic alteration polarizes pro-inflammatory M1 macrophages to anti-inflammatory M2 phenotypes, reducing inflammation and fibrosis in the liver (Fan et al., 2022). Repressing glycolysis and ROS production in CD4^+^ T cells by PGK1 inhibitor NG52 impairs the differentiation of Th17, Th1 and Treg cells, reducing inflammatory injury and subsequent myocardial fibrosis in mice and patients with myocarditis (Lu et al., 2023). D-mannose, a monosaccharide, retards the progression of ulcerative colitis in mice by blocking gluconeogenesis in macrophages and thereby inhibiting succinate-mediated activation of HIF-1α (Torretta et al., 2020).

Antifibrosis research has made potential strides by modifying fatty acid metabolism. Increasing the expression of CPT1 and peroxisomal ACOX1 with C75, a synthetic CPT1 activator, maintains the renal cell viability and inhibits renal fibrosis (Kang et al., 2015). Similarly, C75 reduces the production of collagen, fibronectin and α-SMA by inhibiting FAS, which attenuates the bleomycin-induced pulmonary fibrosis in mice (Jung et al., 2018). Astragaloside IV alleviates palmitate-mediated fibrosis in human glomerular mesangial cells by suppressing CD36 expression (Su et al., 2019). The receptors for FFAs, including GPR40 and GPR84, can also be promising targets for alleviating fibrosis (Gagnon et al., 2018). It has been found that GPR40 inhibits fibrosis, while GPR84 promotes it in a mouse model of renal fibrosis with unilateral ureteral obstruction. TGF-β promotes fibrosis by decreasing GPR40 expression while increasing GPR84 expression (Gagnon et al., 2018). Activating GPR40 with PBI-4050 also inhibits adenine-induced kidney fibrosis in mice (Thibodeau et al., 2019).

The PPAR signaling pathway is crucial for lipid metabolism, including fatty acid uptake and oxidation (Wei et al., 2010; Lakshmi et al., 2017; Cui et al., 2023). PPAR signaling-regulated FAO can promote cell-mediated ECM degradation, which is countered by profibrotic TGF-β (Zhao et al., 2019). As expected, a number of drugs that activate the PPAR signaling pathway exhibit beneficial effect on fibrosis. In a phase 2 clinical trial, the PPARα/δ dual agonist elafibranor improves lipid metabolism and reduces inflammation, which delays liver fibrosis in non-alcoholic steatohepatitis (NASH) patients (Ratziu et al., 2016). Dioscin, a steroidal saponin obtained from the roots of Dioscorea nipponica Makino, can reduce the severity of dextran sulfate sodium (DSS)-induced colitis in mice. This is achieved by increasing the mTORC2/PPARγ-mediated FAO for M2 polarization and decreasing the mTORC1/HIF-1-regulated glycolysis for M1 polarization (Wu et al., 2021). In NASH model mice, the PPARα/γ dual agonist saroglitazar and the PPARα/γ/δ triple agonist lanifibranor are effective in protecting the liver from metabolic disorders and fibrosis (Wettstein et al., 2017; Jain et al., 2018). Apigenin, a flavonoid derived from fruits and vegetables, alleviates inflammation by polarizing M1 macrophages to M2 macrophages (Feng et al., 2016). Apigenin inhibits the NF-κB activation by blocking PPARγ-mediated nuclear translocation of p65, promoting macrophage M2 polarization (Feng et al., 2016). Moreover, apigenin has also been shown to inhibit the expression of SREBP-1c and Fas proteins in alcohol-induced liver injury (Feng et al., 2016; Wang F. et al., 2017). However, one of the major issues with the current application of PPAR agonists for treating fibrosis is the incomplete comprehension of the interactions between PPAR isomers. For example, PPARα agonists can increase serum FFA levels, while the PPARγ agonists can decrease serum FFA levels in a rat model of NASH. Therefore, a better understanding of the interactions between these isoforms is needed to optimize the therapeutic efficacy of targeting PPAR pathway in fibrosis.

Boosting FAO is a strategy for polarizing macrophages toward anti-inflammatory M2 phenotype and preventing fibrosis. Spermidine, a natural polyamine, enhances FAO and OXPHOS fluxes by activating AMPK and HIF-1α during M2 macrophage polarization, which could effectively prevent the progression of colitis to fibrosis (Liu et al., 2020). Didymin, a dietary citrus flavonoid, prevents the progression of ulcerative colitis to fibrosis by enhancing 4 hydroxypropyl-CoA dehydrogenase beta-mediated FAO in macrophages (Lv Q. et al., 2021). Pirfenidone-increased FAO polarizes macrophages to the M2 phenotype and attenuates steatohepatitis in a mouse model (Chen et al., 2019). Fenofibrate attenuates liver fibrosis in mice by activating PPARα and enhancing FAO in macrophages, which drives their polarization to M2 macrophages (Lakhia et al., 2018). MCC950 enhances FAO by inducing CPT1β and CD36 expression in macrophages, and inhibiting GLUT4 to reduce glucose utilization. These metabolic alterations induce the infiltration of M2 macrophages into the sites of cardiac inflammation, reducing myocardial inflammatory injury and preventing myocardial fibrosis (Wang et al., 2022).

Hormone therapy, one of the significant treatments for fibrosis, also plays an important role in regulating cellular respiration and oxidative catabolism. Thyroid hormones are crosstalked with multiple metabolic pathways by regulating PPARs, mTOR and AMPK signaling, which regulate cellular energy metabolism. It has been observed that using thyroid hormones can improve mitochondrial function by increasing PGC1α and PINK1 activity in a mouse bleomycin-induced pulmonary fibrosis model (Wang K. et al., 2018; Yu et al., 2018). The enhanced mitochondrial biosynthetic function and cellular respiration in response to thyroid hormones ultimately alleviate pulmonary fibrosis. Additionally, thyroid hormone can increase miR34a expression to inhibit TGF-β-induced EMT in renal fibrosis (Lu et al., 2013). Thyroid hormone has also been found to bind to thyroid nuclear receptors, which inhibits TGF-β-SMAD signaling and reduces skin fibrosis (Alonso-Merino et al., 2016).

## 5 Conclusion and prospects

Fibrosis is a sequelae of tissue damage which is a potential risk factor for organ dysfunction and even individual death. Although fibrosis poses a great risk to human health, there is still no effective means to prevent and treat fibrosis. The key process leading to tissue fibrosis is the infiltration of immune cells, which create a chronic inflammatory environment at the injury site. Immune cells must undergo metabolic reprogramming to provide sufficient energy and metabolites for their activation and cytokine production, which is well known as immunometabolism. In recent years, an increasing number of studies have shown that immunometabolism contributes to the development and progression of fibrosis. Altered glycolysis, amino acid metabolism, and lipid metabolism induce the formation of inflammatory environment and production of ECM during fibrosis and its related diseases. Notably, these metabolic alterations are also involved in the profibrotic role of TGF-β1 signaling pathway. Insights into the roles of immunometabolism in fibrosis reveal opportunities for potential therapeutic treatment. In particular, the metabolic changes that precede phenotypic alterations in immune cells can be targeted to improve immune cell function, alleviating the progression of inflammation and fibrosis. While many antifibrotic drugs targeting metabolic dysregulation have shown powerful effects in experimental animal models, more clinical studies are needed to test the therapeutic efficacy and adverse effects of these drugs on fibrotic diseases. Moreover, further research is still required to elucidate how the interplay of inflammation and metabolic changes promotes fibrosis, what is the difference in the immunometabolism during physiological tissue repair and pathophysiological fibrotic process, and what is the contributing metabolic pattern in specific tissue, like liver and lung, for fibrotic process, hoping to develop more effective and safer antifibrotic drugs.

## Figures and Tables

**Table:** 

**ACOX**	Acyl-CoA oxidase
**Arg-1**	Arginase-1
**AMPK**	5′-AMP-activated protein kinase
**AMP**	Adenosine monophosphate
**ACLY**	ATP-citrate lyase
**ATP**	Adenosine triphosphate
**AKT**	Protein kinase b
**BCR**	B cell receptor
**BiP**	Immunoglobulin-binding protein
**c-Myc**	Proto-oncogene Myc
**CSR**	Class switching recombination
**CD36**	Cluster of differentiation 36
**CPT1**	Carnitine palmitoyl transferase 1
**CCl4**	Tetrachloride
**DNL**	*De novo* lipogenesis
**DSS**	Dextran sulfate sodium
**DNA**	Deoxyribonucleic acid
**CARKL**	Carbohydrate kinase-like protein
**ECM**	Extracellular matrix
**ETC**	Electron transport chain
**ER**	Endoplasmic reticulum
**EMT**	Epithelial mesenchymal transition
**EndMT**	Endothelial-to-mesenchymal transition
**ERK**	Extracellular signal-regulated kinase
**EGCG**	Epigallocatechin-3-gallate
**FA**	Fatty acid
**FAO**	Fatty acid oxidation
**FAS**	Fatty acid synthesis
**FADH2**	Flavin adenine dinucleotide dihydrogen
**HFXR**	Farnesoid X receptor
**GDH**	Glutamate dehydrogenase
**GLS**	Glutaminase
**GLS1**	Glutaminase 1
**GPR18**	G-protein-coupled receptor 40
**GPR40**	G-protein-coupled receptor 40
**GPR84**	G-protein-coupled receptor 84
**GSK3**	Glycogen synthase kinase-3
**GLP1R**	Glucagon-like peptide 1 receptor
**GLUT1**	Glucose transporter 1
**G6PD**	Glucose-6-phosphate dehydrogenase
**HIF-1**	Hypoxia-inducible factor-1
**HIF-1α**	Hypoxia-inducible factor-1 alpha
**HIF-2α**	Hypoxia-inducible factor-2 alpha
**HMG-CoA**	3-hydroxy-3-methyl-glutaryl-CoA
**HSCs**	Hepatic stellate cells
**HK**	Hexokinase
**H3K27me3**	Trimethylation on histone H3 lysine 27
**IPF**	Idiopathic pulmonary fibrosis
**SSc**	Systemic sclerosis
**IDH**	Isocitrate dehydrogenase
**IFN-γ**	Interferon-gamma
**IL-1β**	Interleukin-1 beta
**IL-2**	Interleukin-2
**IL-4**	Interleukin-4
**IL-4R**	Interleukin-4 receptor
**IL-6**	Interleukin-6
**IL-10**	Interleukin-10
**IL-12**	Interleukin-12
**IL-13**	Interleukin-13
**IL-17**	Interleukin-17
**IL-23**	Interleukin-23
**IGM**	Immunoglobulin M
**JNK**	Jun N-terminal kinase
**JMJD3**	Jumonji C domain-containing demethylase 3
**JAK**	Janus kinase
**LAP**	Latency association protein
**KD**	Ketogenic diet
**LSD1**	Lysine specific demethylase 1
**LPS**	Lipopolysaccharide
**LDH**	Lactate dehydrogenase
**mTORC1**	mTOR complex 1
**mTOR**	mammalian target of rapamycin
**MPCs**	Mesenchymal progenitor cells
**Mpc2**	Mitochondrial pyruvate carrier 2
**MIP-1β**	Macrophage inflammatory protein-1 beta
**MAPK**	Mitogen-activated protein kinase
**MMPs**	Matrix metalloproteinases
**NF-κB**	Nuclear factor kappa-light-chain-enhancer of activated B cells
**NADH**	Nicotinamide adenine dinucleotide hydrogen
**NASH**	Non-alcoholic steatohepatitis
**NO**	Nitric oxide
**OXPHOS**	Oxidative phosphorylation
**PDGF-BB**	Platelet-derived growth factor-BB.
**PPP**	Pentose phosphate pathway
**PGC-1α**	Peroxisome proliferator activated receptor-gamma coactivator-1 alpha
**PGC-1β**	Peroxisome proliferator activated receptor-gamma coactivator-1 beta
**pFOXO1**	Phosphorylated forkhead box protein O1
**PKM2**	Pyruvate kinase muscle isozyme M2
**PPAR**	Peroxisome proliferator-activated receptor
**PFK2**	Phosphofructokinase 2
**PAMP**	Pathogen-associated molecular patterns
**PI3K**	Phosphoinositide 3-kinase
**PCD**	Plasma cell differentiation
**PDK1**	Pyruvate dehydrogenase kinase 1
**PFKFB3**	6-Phosphofructo-2-kinase/fructose 2,6-bisphosphatase 3
**PGE2**	Prostaglandin E2
**PGK1**	Phosphoglycerol kinase 1
**ROS**	Reactive oxygen species
**RA**	Rheumatoid arthritis
**SMAD**	Suppressor of mothers against decapentaplegic
**Smurf2**	SMAD ubiquitination regulatory factor 2
**STAT**	Signal transducer and activator of transcription
**STAT3**	Signal transducer and activator of transcription 3
**STAT6**	Signal transducer and activator of transcription 6
**SDH**	Succinate dehydrogenase
**SLE**	Systemic lupus erythematosus
**Syk**	Spleen tyrosine kinase
**TSP-1**	Thrombospondin 1
**TIMP**	Tissue inhibitor of matrix metalloproteinase
**TD**	T cell-dependent
**TI**	T cell-independent
**TGF-β**	Transforming growth factor-beta
**TGFβR**	TGFβ receptor
**TLR**	Toll like receptor
**TLR4**	Toll like receptor 4
**TCA**	Tricarboxylic acid
**α-KG**	alpha-Ketoglutarate
**TNF-α**	Tumor necrosis factor alpha
**3PO**	3-(3-pyridinyl)−1-(4-pyridinyl)−2-propen-1-one
**2-DG**	2-Deoxyglucose
